# Socioeconomic status and 30-day mortality after minor and major trauma: A retrospective analysis of the Trauma Audit and Research Network (TARN) dataset for England

**DOI:** 10.1371/journal.pone.0210226

**Published:** 2018-12-31

**Authors:** Philip McHale, Daniel Hungerford, David Taylor-Robinson, Thomas Lawrence, Timothy Astles, Ben Morton

**Affiliations:** 1 Department of Public Health and Policy, Institute of Psychology, Health and Society, University of Liverpool, Liverpool, United Kingdom; 2 Institute of Infection and Global Health, University of Liverpool, Liverpool, United Kingdom; 3 Field Epidemiology Service, National Infection Service, Public Health England, Liverpool, United Kingdom; 4 Trauma Audit and Research Network, Manchester Medical Academic Health Sciences Centre, Institute of Population Health, University of Manchester, Salford Royal Hospital, Salford, United Kingdom; 5 Critical Care Department, Aintree University Hospital NHS Foundation Trust, Liverpool, United Kingdom; 6 Clinical Sciences, Liverpool School of Tropical Medicine, Liverpool, United Kingdom; University of Oxford, UNITED KINGDOM

## Abstract

**Introduction:**

Socioeconomic status (SES) is associated with rate and severity of trauma. However, it is unclear whether there is an independent association between SES and mortality after injury. Our aim was to assess the relationship between SES and mortality from trauma.

**Materials and methods:**

We conducted a secondary analysis of the Trauma Audit and Research Network dataset. Participants were patients admitted to NHS hospitals for trauma between January 2015 and December 2015, and resident in England. Analyses used multivariate logistic regression with thirty-day mortality as the main outcome. Co-variates include SES derived from area-level deprivation, age, injury severity and comorbidity. All analyses were stratified into minor and major trauma.

**Results:**

There were 48,652 admissions (68% for minor injury, ISS<15) included, and 3,792 deaths. Thirty-day mortality was 10% for patients over 85 with minor trauma, which was higher than major trauma for all age groups under 65. Deprivation was not significantly associated with major trauma mortality. For minor trauma, patients older than 40 had significantly higher aORs than the 0–15 age group. Both the most and second most deprived had significantly higher aORs (1.35 and 1.28 respectively).

**Conclusions:**

This study provides evidence of an independent relationship between SES and mortality after minor trauma, but not for major trauma. Our results identify that, for less severe trauma, older patients and patients with low SES with have an increased risk of 30-day mortality. Policy makers and service providers should consider extending the provision of ‘major trauma’ healthcare delivery to this at-risk population.

## Introduction

Traumatic injury is a major public health problem. In 2013 there were an estimated 973 million episodes that required healthcare, and 4.8 million deaths worldwide [1]. In England and Wales, trauma causes 16,000 deaths each year, with many survivors left with severe and long lasting disability [2]. In recent years, systems improvements such as the establishment of major trauma networks in England and advances in care have promoted improved outcomes, but major trauma remains a significant issue. In 2010, the UK National Audit Office estimated the economic losses associated with major trauma in England was between 3.3 and 3.7 billion pounds [3], and trauma is estimated to cost Clinical Commissioning Groups £1.53 billion [4].

There is substantial evidence of an association between socioeconomic status (SES) and trauma in different settings and across age groups. In the UK, lower SES is associated with increased rates of injuries in children [5], rates of house fire [6], and twice the rate of traumatic injury in adults [7]. Similar patterns are seen internationally for children [8–10], and adults [11–13]. However, the relationship between trauma mortality and SES is less clear. Low income has been linked to higher mortality from trauma in the USA after adjustment for race, comorbidities and injury severity [14]. However, the effect of differential access to medical care based on SES seen in non-universal healthcare systems may have influenced these findings. Conversely, a recent registry based Scottish (universally free healthcare) study found that SES was not independently associated with trauma mortality [15].

The aim of this study is to determine the relationship between SES and trauma mortality, and how this varies by injury severity. Addressing these questions is important for public health policymakers and clinicians designing policies to address potential inequalities in trauma outcomes.

## Materials and methods

We conducted a retrospective analysis of the TARN database, a clinical registry that collects data from all “trauma-receiving” hospitals in England for patients with traumatic injuries. The best practice tariff for major trauma centres promotes timely entry of clinical data to TARN [16]. TARN has PIAG Section 60 approval for research using the data that it holds. We split the analysis into minor and major trauma (ISS of less than and greater than 15 respectively). The trauma network system in England is predominantly focused on the most severely injured patients by concentrating resources in Major Trauma Centres and caring for the highest acuity patients at these hospitals [17]. Therefore, minor and major trauma patients are treated using distinct clinical pathways, and are analysed as such. We used national data from NHS hospitals (universally free healthcare) in England from the Trauma Audit and Research Network (TARN) to examine this potential effect.

### Participants

We included all individuals who were admitted to “trauma-receiving” hospitals secondary to trauma and treated as an in-patient for 3 or more days, admitted to critical care units or died in hospital. Isolated closed limb fractures and neck of femur fractures in patients over 65 are excluded from TARN. Data collection period: 1^st^ January 2015 and 31^st^ December 2015 and limited to patient’s resident in England. Data include patient age and sex, postcode sector, mode of injury, injury severity score (ISS), comorbidity and mortality at 30, 90 and 180 days.

### Variables

The primary outcome was 30-day mortality. Thirty-day mortality is determined by referencing hospital records for in-patients and via linkage to Office for National Statistics data for those who have been discharged. We selected 30-day mortality as the primary outcome variable as primary mortality diagnoses at 90 and 180 days are increasingly less likely to be caused by the index trauma episode.

The primary predictor variable was SES based on deprivation of area of residence. This study assigned a standardised measure of deprivation to each record in TARN, using the English indices of deprivation 2015, Index of Multiple Deprivation (IMD) [18]. The indices are derived from census and local administrative data and used to construct seven domains of deprivation: income, employment, health and disability, education skill and training, barriers to housing and other services, crime, and living environment. The IMD is a weighted score of the seven domains, and a robust and commonly used measure of deprivation in England. The IMD is available at Lower Super Output Area (LSOA) level, a small geographical boundary containing approximately 1500 persons. Within TARN each record has a geographical indicator, the postcode-sector. To assign the Indices of Deprivation to each record we calculated a synthetic domain score and IMD score for each postcode sector in England by weighting LSOA level deprivation scores based on the geographical contribution of each LSOA to each postcode sector [18]. The resulting synthetic scores were then grouped into country specific national quintiles of deprivation for the IMD, where quintile 1 is the most deprived and quintile 5 the least deprived [19]. This is an established method of IMD estimation that has been used previously [20].

Covariates included age group, sex, ISS category and comorbidity. Age was grouped into 0 to 15 years old, 16 to 24, 25 to 39, 40 to 64, 65 to 84, and 85 and older. ISS was split into four categories, two in major trauma (ISS 16 to 24 and greater than 24), and two in minor trauma (ISS less than 9 and 9 to 15). These are established cut-offs [21,22], and their usefulness derives from patients with isolated injuries of increasing severity appearing in different groups. Comorbidity is calculated by TARN using the pre-existing medical conditions (PMC) score, modified from the Charlson Comorbidity Index. Each comorbid condition is assigned a weight, based on the impact of the condition on outcome, with higher values assigned to more severe conditions. Weights are then summed for each case [23,24]. The scores were categorised as 0, 1 to 5, 6 to 10 and greater than 10 as per TARN criteria.

### Statistical analysis

Data were analysed using Stata V13 (StataCorp, Stata Statistical Software: Release 13, College Station, Texas, USA). All analyses were run separately for both minor and major trauma. Absolute 30-day mortality, and proportion, were determined for each age group, sex, ISS categorisation, comorbidity group and IMD quintile. Cross-tables of IMD quintile by other variables with Chi squared tests are included in the supporting information (S1 Table). Univariate logistic regression models with 30–day mortality as the outcome measure were ran for age group, sex, ISS categorisation, comorbidity and IMD quintile. A multivariable logistic regression model was run with 30–day mortality as the outcome measure and all variables included in univariate regression input. Model fitting was based on variables identified as part of *a priori* statistical plan and used a categorical variable for socioeconomic deprivation (reference group: quintile 5), sex (reference group: female), age group (reference group: 0–15 years), and ISS category (reference group for minor: ISS < 9; reference group for major: ISS 16–24). Additional multivariate regression models with deprivation included as a continuous variable (IMD score) are included in the supporting information (S2 Table).

Sensitivity analyses were run using 90-day and 180-day mortality as the outcome variable for the multivariate logistic regression models. The results for the sensitivity analyses are included in the supplementary appendix (S3 and S4 Tables).

## Results

There were 52,422 trauma admissions to hospital included in the TARN dataset. There were 1,414 admissions excluded because no postcode was available or patients were non-English residents, and one exclusion due to unreliable age data. A further 2,220 were removed because comorbidity data was missing, and 129 were removed because no deprivation score could be assigned to the partial postcode. The final analysis included 48,658 admissions and 3,792 deaths at 30 days, and 68% (n = 32,931) of these admissions were for minor injury.

Table 1 shows the sample characteristics split between minor and major trauma. Over half of minor trauma occurred in patients over 65 years of age, and 29% occurred in those aged 40–64 and approximately 10% came from patients under 24 years of age. Females were responsible for 52% of minor trauma admissions, and 68% of all minor trauma admissions were for severity 9 to 15. Almost half of admissions had a comorbidity score of 0, and 3% had the highest score of more than 10. A social gradient was observed, with 22.9% of admissions from the most deprived compared with 18% from the least deprived areas. Overall, 4% of minor trauma admissions resulted in death at 30 days.

**Table 1:** Characteristics of study population for minor and major trauma.

		Minor		Major	
		n	%	n	%
Age Group	0–15	1,547	4.7	687	4.4
	16–24	1,908	5.8	1,412	9.0
	25–39	2,913	8.8	1,946	12.4
	40–64	9,571	29.1	4,076	25.9
	65–84	10,066	30.6	4,857	30.9
	85+	6,926	21.0	2,749	17.5
Sex	Female	16,968	51.5	5,543	35.3
	Male	15,963	48.5	10,184	64.8
Injury Severity	ISS <9	10,629	32.3		
	ISS 9–15	22,302	67.7		
	ISS 16–24			8,196	52.1
	ISS >24			7,531	47.9
30-Day Mortality	No	31,607	96.0	13,259	84.3
	Yes	1,324	4.0	2,468	15.7
Comorbidity score PMC	0	15,716	47.7	7,198	45.8
	1 to 5	12,706	38.6	6,105	38.8
	6 to 10	3,611	11.0	1,937	12.3
	>10	898	2.7	487	3.1
IMD Quintile	1- most deprived	7,540	22.9	3,657	23.3
	2	7,095	21.5	3,321	21.1
	3	6,530	19.8	3,112	19.8
	4	5,841	17.7	2,796	17.8
	5- least deprived	5,925	18.0	2,841	18.1

PMC- Comorbidity score; ISS- Injury Severity Score; IMD- Index of Multiple Deprivation.

Major trauma admissions were most common from patients in the 40–64 and 65–84 age groups (26% and 31% respectively) but patients from 25–39 and 16–24 age groups made up a larger proportion of admissions (12% and 9% respectively) compared to minor trauma. Overall, 65% of major trauma admissions were male. Overall, 46% of admissions had a comorbidity score of 0, with 3% of patients suffering the highest score (>10). Again, a social gradient was observed, with 23% of admissions from the most deprived compared with 18% from the least deprived areas. Overall, 15% of major trauma admissions resulted in death at 30 days. For both major and minor trauma, significant associations were shown between IMD quintile and age group, sex and comorbidity score. There was a significant association between IMD quintile and thirty-day mortality for major trauma, and between IMD quintile and injury severity for minor trauma (S1 Table).

Table 2 shows the 30-day mortality percentage for each group for minor and major trauma. The mortality proportion increased with age for both, with 10% in patients aged 85 plus for minor trauma and 30% for major trauma. The mortality proportion for 85 plus was higher in minor trauma than those for all patients under 65 for major trauma. The most severe trauma (ISS>24) suffered higher mortality than other ISS categories. Large mortality increases were seen as comorbidity score increased for both minor and major trauma. The relative increase was approximately 14 for minor trauma (1% at score of 0 to 14% at > 10) compared to 3.74 for major trauma (8% to 32%).

**Table 2:** Percentage who died at 30 days in each group, split into minor and major trauma.

		Minor		Major	
		n	%	n	%
Age Group	0–15	4	0.3	35	5.1
	16–24	4	0.2	112	7.9
	25–39	9	0.3	149	7.7
	40–64	121	1.3	381	9.3
	65–84	487	4.8	972	20.0
	85+	699	10.1	819	29.8
Sex	Female	748	4.4	956	17.2
	Male	576	3.6	1,512	14.8
Injury Severity	ISS <9	379	3.6		
	ISS 9–15	945	4.2		
	ISS 16–24			608	7.4
	ISS >24			1,860	24.7
Comorbidity score PMC	0	174	1.1	611	8.5
	1 to 5	635	5.0	1,161	19.0
	6 to 10	388	10.7	541	27.9
	>10	127	14.1	155	31.8
IMD Quintile	1- most deprived	284	3.8	502	13.7
	2	287	4.0	482	14.5
	3	271	4.2	517	16.6
	4	245	4.2	499	17.8
	5- least deprived	237	4.0	468	16.5

PMC- Comorbidity score; ISS- Injury Severity Score; IMD- Index of Multiple Deprivation.

**Fig 1:**
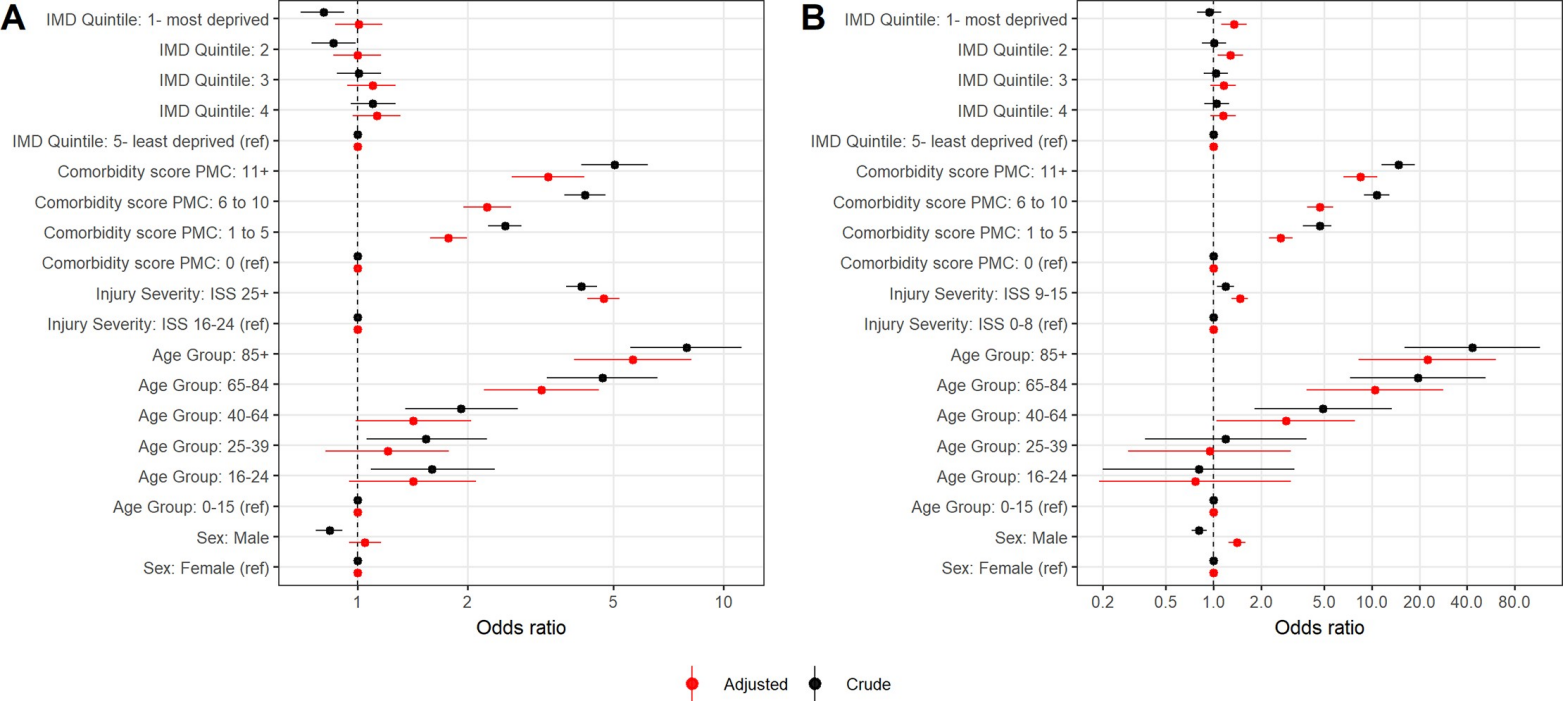
Univariate and multivariate logistic regression models 30-day mortality. Covariates are IMD quintile, age group, sex and ISS categorisation, for A—major trauma and B—minor trauma.

For major trauma, univariate analysis demonstrated that all age groups had significantly higher odds of 30-day mortality compared to patients aged 0–15, with the highest odds for the 85 plus age group (OR 7.91, 95% CI 5.57–11.21; Fig 1). However, only the 65 to 84 and 85 plus age groups had significantly higher adjusted odds ratios (aORs 3.18 and 5.64 respectively). Neither sex, nor deprivation were significantly associated with mortality in either model. All comorbidity groups had higher unadjusted and adjusted odds of mortality compared with a comorbidity score of 0. The highest OR and aOR was for a comorbidity score of over 10 (5.03 and 3.32 respectively). The odds of mortality for ISS of greater than 24 compared to 16 to 24 was 4.09 unadjusted (95% CI 3.71–4.51) and 4.70 adjusted (95% CI 4.24–5.20).

For minor trauma, patients more than 40 years of age had significantly higher odds of mortality than the 0–15 age group, both for univariate and multivariate models. The odds were highest for the 85 plus age group, with an OR of 43.30 (95% CI 16.18–115.88) and aOR of 22.42 (95% CI 8.29–60.63). Males had significantly higher odds than females in the multivariate model only (aOR 1.41, 95% CI 1.25–1.59). All comorbidity groups had significantly higher unadjusted and adjusted odds of mortality compared with a comorbidity score of 0. The highest OR and aOR was for a comorbidity score of over 10 (14.71 and 8.48 respectively). The odds of mortality for ISS of 9 to 15 compared to less than 9 was 1.20 unadjusted (95% CI 1.06–1.35) and 1.47 adjusted (95% CI 1.30–1.66). Unadjusted OR were not significant for deprivation, however aOR for most deprived (1.35, 95% CI 1.12–1.62) and second most deprived (1.28, 95% CI 1.07–1.54) were both significant compared to the least deprived quintile.

Sensitivity analyses using 90-day and 180-day mortality as the outcome variable have broadly similar patterns as using 30-day mortality (see S3 and S4 Tables). For major trauma, there were no significant findings for deprivation and the patterns seen for other covariates remained broadly similar. For minor trauma, increasing the mortality horizon moderated the odds related to deprivation. The aORs remained significantly higher for the most deprived compared to the least deprived at both 90 and 180 days (1.27 and 1.26 respectively), however the second most deprived quintile was no longer significant at 180 days (aOR 1.12, 95% CI 0.98–1.27). All other covariate patterns remained broadly similar.

## Discussion

In this large, retrospective analysis of national data of hospital admissions due to injury in England we show that for major trauma, deprivation did not have an independent relationship with 30-day mortality. Conversely, after minor trauma there was significantly higher 30-day mortality for people living in more disadvantaged areas.

For minor trauma, there was a clear social gradient for mortality, with the lowest SES associated with highest odds of mortality. The lack of specialist care for non-major trauma may explain the increased odds of mortality seen in more disadvantaged groups. In England, all healthcare is free at point of care through the NHS and the majority of major trauma is attended to by a trauma team at specialist trauma centres [17]. This involves a team of highly specialised clinicians who provide high quality care for patients. Evidence suggests that models such as this have led to improvements in outcomes from severe trauma [25], and better access to healthcare is associated reductions in inequalities of mortality across Europe [26]. It is plausible that this specialist care for major trauma and the large effect of injury severity on mortality for major trauma masks the relatively small effect that SES may have on mortality from major trauma, in contrast to less severe injuries. This lack of specialist care for non-major trauma may lead to the ‘inverse care’ effect; despite having increased need of care, individuals from low SES are less likely to receive it, and therefore are at increased risk of mortality. Despite the availability of universal healthcare, the inverse care law remains applicable in the UK [27]. Another potential mechanism for this association could be worse underlying health of people from lower SES meaning they are less able to survive the insult of minor trauma. We have controlled for comorbidities in these analyses in an attempt to offset this mechanism.

The observed relationship between covariates and mortality for minor trauma is suggestive that different groups are particularly vulnerable to mortality. High aORs are seen for older age groups and for increasing levels of comorbidity which may highlight these groups as highly vulnerable after minor trauma. An alternative interpretation is that these results suggest that these groups are at baseline higher risk on mortality and that the presence of minor trauma is incidental. However, we suggest that using 30-day mortality as our primary outcome measure increases the likelihood that mortality was directly attributable to the trauma episode.

Previous studies have provided contradictory evidence regarding the association of SES on mortality in trauma. Despite some previous studies suggesting there is no association between SES and mortality [15,28], our study shows SES is associated with minor trauma mortality [14], but not for major trauma. This has implications for both public health and clinical practice. For public health, preventative work should consider the importance of less severe injuries in older age groups and in more disadvantaged communities, and extra service provision may be required for rehabilitation or follow-up care. Our results demonstrate that older patients with minor trauma had similar mortality to younger age groups with major trauma. This raises the possibility that advanced age should be used as a criterion for trauma team activation (65 years old and above) [29]. There may also be an important role for ortho-geriatricians in the elderly patients with minor trauma, as has become commonplace for elderly patients who sustain neck of femur fractures in the NHS (not included within this dataset).

There are a number of strengths to our study. The use of the TARN database for England means near complete capture of major traumatic injuries, as all emergency admissions will attend an NHS hospital. Thus, the analysis should be fully representative of the major trauma population during this time period. Additionally, the universal coverage provided by the NHS attenuates, but does not completely remove, the effect of differential healthcare access, an issue that has influenced prior studies [28]. The inclusion of a comorbidity measure strengthens the results, as our findings suggest that comorbidity does not confound the observed mortality—SES relationship. Finally, data availability and coverage has allowed us to analyse the impact of non-major trauma, including minor injuries, and we have examined this group as a separate patient population to major injury due to the different clinical pathways of care they follow [17].

A number of limitations exist. As only partial postcodes were available, the area-based measure of deprivation is less accurate than it would be if full postcode was available, particularly when compared to previous studies on the topic [15]. Conversely, evidence from the US uses ZIP-code linked data [14,28], which includes approximately 30,000 people [30], and our area-based measure includes a median of 6,799 people. Therefore, our methodology may risk ecological fallacy when interpreting results. Another limitation is the use of mortality as the primary outcome variable. Mortality is only one negative outcome for trauma, and the study was unable to determine the relationship between socioeconomic status and other negative sequelae, such as disability. The inclusion of only trauma with a length of stay of three or more days could impact on the generalisability of our findings. There will be a cohort of injuries that are excluded from our analysis because they are treated through a short hospital stay, in the emergency department, in primary care, or through self-care. This is unlikely to impact the major trauma analysis, however for minor trauma there may be a cohort of excluded cases who are at low risk of the mortality outcome, introducing a potential source of bias.

In conclusion, this study provides evidence of an independent relationship between SES and mortality after minor trauma, but not for major trauma. Major trauma centres preferentially focus resources on patients with major trauma. Our results identify a pertinent subset of patients with less severe trauma with increased risk of 30-day mortality. Policy makers and service providers should consider extending the provision of ‘major trauma’ healthcare delivery to this at-risk population. Our findings indicate that further targeted resource investment in preventative practices for older patients living in deprived areas may be appropriate to reduce mortality after trauma. Further research is needed to explore how SES impacts other negative outcomes from trauma, such as longer-term morbidity and disability.

## Supporting information

S1 Table **Characteristics of sample by IMD quintile with Chi squared test statistics, for: A–minor trauma, and B–major trauma.** PMC- Comorbidity score; ISS- Injury Severity Score; IMD- Index of Multiple Deprivation.
(DOCX)

S2 Table Multivariate logistic regression models 30-day mortality by IMD score, age group, sex and ISS categorisation, for major and minor trauma. PMC- Comorbidity score; ISS- Injury Severity Score; IMD- Index of Multiple Deprivation.
(DOCX)

S3 Table Multivariate logistic regression models 90-day mortality by IMD quintile, age group, sex and ISS categorisation, for minor and major trauma. PMC- Comorbidity score; ISS- Injury Severity Score; IMD- Index of Multiple Deprivation.
(DOCX)

S4 Table Multivariate logistic regression models 180-day mortality by IMD quintile, age group, sex and ISS categorisation, for minor and major trauma. PMC- Comorbidity score; ISS- Injury Severity Score; IMD- Index of Multiple Deprivation.
(DOCX)

## References

[1] Haagsma JA, Graetz N, Bolliger I, Naghavi M, Higashi H, Mullany EC, et al The global burden of injury: incidence, mortality, disability-adjusted life years and time trends from the Global Burden of Disease study 2013. Inj Prev. 2016 2 1;22(1):3 10.1136/injuryprev-2015-041616 26635210 PMC4752630

[2] The Trauma Audit and Research Network. TARN—Home [Internet]. [cited 2017 Nov 6]. Available from: https://www.tarn.ac.uk/

[3] National Audit Office. Major Trauma Care in England. London: National Audit Office; 2010.

[4] Kellezi B, Baines DL, Coupland C, Beckett K, Barnes J, Sleney J, et al The impact of injuries on health service resource use and costs in primary and secondary care in the English NHS. J Public Health. 2016 12 2;38(4):e464–71. 10.1093/pubmed/fdv173 28158513

[5] Pearce A, Li L, Abbas J, Ferguson B, Graham H, Law C. Does the home environment influence inequalities in unintentional injury in early childhood? Findings from the UK Millennium Cohort Study. J Epidemiol Community Health. 2012 2 1;66(2):181 10.1136/jech.2011.139626 22003079

[6] Mulvaney C, Kendrick D, Towner E, Brussoni M, Hayes M, Powell J, et al Fatal and non-fatal fire injuries in England 1995–2004: time trends and inequalities by age, sex and area deprivation. J Public Health. 2009;31(1):154–61. 10.1093/pubmed/fdn103 19074453

[7] Corfield A, Pell J, MacKay D. Nested: National trauma registry study of deprivation. Ann Emerg Med. 2015;66(4).

[8] Laursen B, Nielsen JW. Influence of sociodemographic factors on the risk of unintentional childhood home injuries. Eur J Public Health. 2008;18(4):366–70. 10.1093/eurpub/ckn034 18515863

[9] Laflamme L, Hasselberg M, Reimers A-M, Cavalini LT, Ponce de Leon A. Social determinants of child and adolescent traffic-related and intentional injuries: a multilevel study in Stockholm County. Soc Sci Med 1982. 2009;68(10):1826–34. 10.1016/j.socscimed.2009.02.050 19346046

[10] Nyberg C, Schyllander J, Stark Ekman D, Janson S. Socio-economic risk factors for injuries in Swedish children and adolescents: a national study over 15 years. Glob Public Health. 2012;7(10):1170–84. 10.1080/17441692.2012.736172 23152975

[11] Gotsens M, Marí-Dell’Olmo M, Pérez K, Palència L, Martinez-Beneito M-A, Rodríguez-Sanz M, et al Socioeconomic inequalities in injury mortality in small areas of 15 European cities. Health Place. 2013;24:165–72. 10.1016/j.healthplace.2013.09.003 24112963

[12] Lawson F, Schuurman N, Amram O, Nathens AB. A geospatial analysis of the relationship between neighbourhood socioeconomic status and adult severe injury in Greater Vancouver. Inj Prev 1353–8047. 2015;21(4):260–5. 10.1136/injuryprev-2014-041437 25694418 PMC4518736

[13] Lee J, Lee W-Y, Noh M, Khang Y-H. Does a geographical context of deprivation affect differences in injury mortality? A multilevel analysis in South Korean adults residing in metropolitan cities. J Epidemiol Community Health. 2014;68(5):457–65. 10.1136/jech-2013-203082 24550434

[14] Ali MT, Hui X, Hashmi ZG, Dhiman N, Scott VK, Efron DT, et al Socioeconomic disparity in inpatient mortality after traumatic injury in adults. Surgery. 2013;154(3):461–7. 10.1016/j.surg.2013.05.036 23972652 PMC3989530

[15] Corfield A, MacKay D, Pell J. Association between trauma and socioeconomic deprivation: a registry-based, Scotland-wide retrospective cohort study of 9,238 patients. Scand J Trauma Resusc Emerg Med. 2016;24(1):90. 27388437 10.1186/s13049-016-0275-7 PMC4937548

[16] NICE. Major trauma: service delivery [Internet]. 2016 [cited 2018 Jan 18]. Available from: https://www.nice.org.uk/guidance/ng40/chapter/recommendations-for-research

[17] NHS Choices. Major trauma services—Emergency and urgent care [Internet]. 2017 [cited 2017 Nov 25]. Available from: https://www.nhs.uk/NHSEngland/AboutNHSservices/Emergencyandurgentcareservices/Pages/Majortraumaservices.aspx

[18] Gill B. The English Indices of Deprivation 2015. London: Department for Communities and Local Government; 2015.

[19] UK Data Service Census Support. GeoConvert: Frequently Asked Questions [Internet]. 2015 [cited 2018 Jan 18]. Available from: http://geoconvert.mimas.ac.uk/help/faq.html

[20] Hungerford D, Vivancos R, Read JM, Iturriza-Gόmara M, French N, Cunliffe NA. Rotavirus vaccine impact and socioeconomic deprivation: an interrupted time-series analysis of gastrointestinal disease outcomes across primary and secondary care in the UK. BMC Med. 2018 1 29;16(1):10 10.1186/s12916-017-0989-z 29375036 PMC5787923

[21] Stevenson M, Segui-Gomez M, Lescohier I, Di Scala C, McDonald-Smith G. An overview of the injury severity score and the new injury severity score. Inj Prev. 2001 3 1;7(1):10 10.1136/ip.7.1.10 11289527 PMC1730702

[22] Palmer C. Major Trauma and the Injury Severity Score—Where Should We Set the Bar? Annu Proc Assoc Adv Automot Med. 2007;51:13–29. 18184482 PMC3217501

[23] The Trauma Audit and Research Network. The TARN Probability of Survival Model [Internet]. [cited 2018 Apr 26]. Available from: https://www.tarn.ac.uk/Content.aspx?c=3515

[24] Bouamra O, Jacques R, Edwards A, Yates DW, Lawrence T, Jenks T, et al Prediction modelling for trauma using comorbidity and ‘true’ 30-day outcome. Emerg Med J. 2015 12 1;32(12):933 10.1136/emermed-2015-205176 26493123

[25] Moran CG, Lecky F, Bouamra O, Lawrence T, Edwards A, Woodford M, et al Changing the System—Major Trauma Patients and Their Outcomes in the NHS (England) 2008–17. EClinicalMedicine. 2018 8 1;2:13–21. 10.1016/j.eclinm.2018.07.001 PMC6537569 31193723

[26] Mackenbach JP, Hu Y, Artnik B, Bopp M, Costa G, Kalediene R, et al Trends In Inequalities In Mortality Amenable To Health Care In 17 European Countries. Health Aff (Millwood). 2017 6 1;36(6):1110–8. 28583971 10.1377/hlthaff.2016.1674

[27] Socialist Health Association. A Review of the Literature: Does the Inverse Care Law Still Apply Today? [Internet]. Socialist Health Association. 2010 [cited 2018 Dec 10]. Available from: https://www.sochealth.co.uk/national-health-service/public-health-and-wellbeing/poverty-and-inequality/the-inverse-care-law/a-review-of-the-literature-does-the-inverse-care-law-still-apply-today/

[28] Mikhail JN. The Association of Race, Socioeconomic Status, and Insurance on Trauma Mortality. J Trauma Nurs. 2016 12 11;23(6):347–56. 10.1097/JTN.0000000000000246 27828890

[29] Demetriades D, Sava J, Alo K, Newton E, Velmahos GC, Murray JA, et al Old Age as a Criterion for Trauma Team Activation. J Trauma Acute Care Surg. 2001;51(4):754–7. 10.1097/00005373-200110000-00022 11586171

[30] Krieger N, Waterman P, Chen JT, Soobader M-J, Subramanian SV, Carson R. Zip Code Caveat: Bias Due to Spatiotemporal Mismatches Between Zip Codes and US Census–Defined Geographic Areas—The Public Health Disparities Geocoding Project. Am J Public Health. 2002 7;92(7):1100–2. 12084688 10.2105/ajph.92.7.1100 PMC1447194

