# Supplementary material for: Socioeconomic status and 30-day mortality after minor and major trauma: A retrospective analysis of the Trauma Audit and Research Network (TARN) dataset for England
Source: PLoS One. 2018 Dec 31;13(12):e0210226. doi: 10.1371/journal.pone.0210226 (PMC6312286; doi:10.1371/journal.pone.0210226)
Supplement: S1 Table — Characteristics of sample by IMD quintile with Chi squared test statistics, for: A–minor trauma, and B–major trauma. PMC- Comorbidity score; ISS- Injury Severity Score; IMD- Index of Multiple Deprivation. (DOCX) [file pone.0210226.s001.docx]

**S1 Table.**

**A**

|  |  | IMD Quintile | | | | |  |  |
| --- | --- | --- | --- | --- | --- | --- | --- | --- |
|  |  | 1- most deprived | 2 | 3 | 4 | 5- least deprived | Chi^2^ | p-value |
| Age Group | 0-15 | 500 | 352 | 259 | 212 | 224 |  |  |
|  | 16-24 | 579 | 446 | 338 | 311 | 234 |  |  |
|  | 25-39 | 873 | 729 | 527 | 428 | 356 |  |  |
|  | 40-64 | 2,509 | 2,155 | 1,815 | 1,606 | 1,486 |  |  |
|  | 65-84 | 1,997 | 2,066 | 2,125 | 1,882 | 1,996 |  |  |
|  | 85+ | 1,082 | 1,347 | 1,466 | 1,402 | 1,629 | 829.5 | <0.001 |
| Sex | Female | 3,401 | 3,526 | 3,447 | 3,188 | 3,406 |  |  |
|  | Male | 4,139 | 3,569 | 3,083 | 2,653 | 2,519 | 244.1 | <0.001 |
| Injury Severity | ISS <9 | 2,297 | 2,296 | 2,157 | 1,910 | 1,969 |  |  |
|  | ISS 9-15 | 5,243 | 4,799 | 4,373 | 3,931 | 3,956 | 16.0 | 0.003 |
| Comorbidity score PMC | 0 | 3,592 | 3,425 | 3,148 | 2,839 | 2,712 |  |  |
|  | 1 to 5 | 2,949 | 2,744 | 2,461 | 2,180 | 2,372 |  |  |
|  | 6 to 10 | 758 | 733 | 744 | 685 | 691 |  |  |
|  | >10 | 241 | 193 | 177 | 137 | 150 | 39.2 | <0.001 |
| 30-Day Mortality | No | 7,256 | 6,808 | 6,259 | 5,596 | 5,688 |  |  |
|  | Yes | 284 | 287 | 271 | 245 | 237 | 2.0 | 0.732 |

**B**

|  |  | IMD Quintile | | | | |  |  |
| --- | --- | --- | --- | --- | --- | --- | --- | --- |
|  |  | 1- most deprived | 2 | 3 | 4 | 5- least deprived | Chi^2^ | p-value |
| Age Group | 0-15 | 216 | 146 | 122 | 113 | 90 |  |  |
|  | 16-24 | 423 | 300 | 272 | 207 | 210 |  |  |
|  | 25-39 | 623 | 483 | 374 | 263 | 203 |  |  |
|  | 40-64 | 1,051 | 902 | 766 | 676 | 681 |  |  |
|  | 65-84 | 882 | 966 | 1,010 | 985 | 1,014 |  |  |
|  | 85+ | 462 | 524 | 568 | 552 | 643 | 462.6 | <0.001 |
| Sex | Female | 1,087 | 1,124 | 1,143 | 1,059 | 1,130 |  |  |
|  | Male | 2,570 | 2,197 | 1,969 | 1,737 | 1,711 | 88.7 | <0.001 |
| Injury Severity | ISS 16-24 | 1,870 | 1,756 | 1,625 | 1,418 | 1,527 |  |  |
|  | ISS >24 | 1,787 | 1,565 | 1,487 | 1,378 | 1,314 | 7.4 | 0.115 |
| Comorbidity score PMC | 0 | 1,757 | 1,547 | 1,390 | 1,254 | 1,250 |  |  |
|  | 1 to 5 | 1,369 | 1,307 | 1,233 | 1,065 | 1,131 |  |  |
|  | 6 to 10 | 412 | 363 | 382 | 400 | 380 |  |  |
|  | >10 | 119 | 104 | 107 | 77 | 80 | 34.9 | <0.001 |
| 30-Day Mortality | No | 3,155 | 2,839 | 2,595 | 2,297 | 2,373 |  |  |
|  | Yes | 502 | 482 | 517 | 499 | 468 | 27.3 | <0.001 |
