# Supplementary material for: Socioeconomic status and 30-day mortality after minor and major trauma: A retrospective analysis of the Trauma Audit and Research Network (TARN) dataset for England
Source: PLoS One. 2018 Dec 31;13(12):e0210226. doi: 10.1371/journal.pone.0210226 (PMC6312286; doi:10.1371/journal.pone.0210226)
Supplement: S2 Table — PMC- Comorbidity score; ISS- Injury Severity Score; IMD- Index of Multiple Deprivation. (DOCX) [file pone.0210226.s002.docx]

**S2 Table.**

|  |  | Minor | | Major | |
| --- | --- | --- | --- | --- | --- |
|  |  | OR | 95% CI | AOR | 95% CI |
| Age Group | 0-15 | REF | - | REF | - |
|  | 16-24 | 0.78 | 0.19-3.12 | 1.41 | 0.94-2.09 |
|  | 25-39 | 0.95 | 0.29-3.11 | 1.20 | 0.82-1.77 |
|  | 40-64 | 2.88 | 1.06-7.84 | 1.41 | 0.98-2.03 |
|  | 65-84 | 10.55 | 3.91-28.52 | 3.15 | 2.19-4.51 |
|  | 85+ | 22.59 | 8.35-61.11 | 5.56 | 3..85-8.03 |
| Sex | Female | REF | - | REF | - |
|  | Male | 1.41 | 1.25-1.59 | 1.05 | 0.95-1.16 |
| Injury Severity | ISS <9 | REF | - |  |  |
|  | ISS 9-15 | 1.47 | 1.30-1.66 |  |  |
|  | ISS 16-24 |  |  | REF | - |
|  | ISS >24 |  |  | 4.70 | 4.24-5.21 |
| Comorbidity score PMC | 0 | REF | - | REF | - |
|  | 1 to 5 | 2.66 | 2.24-3.17 | 1.78 | 1.58-2.00 |
|  | 6 to 10 | 4.71 | 3.90-5.70 | 2.27 | 1.96-2.64 |
|  | >10 | 8.43 | 6.58-10.80 | 3.34 | 2.65-4.19 |
| IMD Score | | 1.01 | 1.00-1.01 | 1.00 | 0.99-1.00 |
