# Supplementary material for: Socioeconomic status and 30-day mortality after minor and major trauma: A retrospective analysis of the Trauma Audit and Research Network (TARN) dataset for England
Source: PLoS One. 2018 Dec 31;13(12):e0210226. doi: 10.1371/journal.pone.0210226 (PMC6312286; doi:10.1371/journal.pone.0210226)
Supplement: S3 Table — PMC- Comorbidity score; ISS- Injury Severity Score; IMD- Index of Multiple Deprivation. (DOCX) [file pone.0210226.s003.docx]

**S3 Table.**

|  |  | Minor | | Major | |
| --- | --- | --- | --- | --- | --- |
|  |  | OR | 95% CI | AOR | 95% CI |
| Age Group | 0-15 | REF | - | REF | - |
|  | 16-24 | 0.96 | 0.26-3.58 | 1.40 | 0.95-2.07 |
|  | 25-39 | 0.96 | 0.29-3.12 | 1.20 | 0.82-1.76 |
|  | 40-64 | 5.09 | 1.88-13.76 | 1.43 | 1.00-2.05 |
|  | 65-84 | 17.62 | 6.55-47.37 | 3.52 | 2.47-5.01 |
|  | 85+ | 43.58 | 16.20-117.21 | 7.06 | 4.92-10.11 |
| Sex | Female | REF | - | REF | - |
|  | Male | 1.31 | 1.19-1.44 | 1.01 | 0.92-1.11 |
| Injury Severity | ISS <9 | REF | - | REF | - |
|  | ISS 9-15 | 1.27 | 1.15-1.40 | 3.79 | 3.45-4.17 |
| Comorbidity score PMC | 0 | REF | - | REF | - |
|  | 1 to 5 | 2.52 | 2.21-2.87 | 1.84 | 1.65-2.06 |
|  | 6 to 10 | 4.21 | 3.63-4.87 | 2.63 | 2.28-3.03 |
|  | >10 | 7.76 | 6.33-9.51 | 3.88 | 3.11-4.83 |
| IMD Quintile | 1- most deprived | 1.27 | 1.10-1.46 | 1.04 | 0.90-1.20 |
|  | 2 | 1.16 | 1.00-1.34 | 1.04 | 0.91-1.20 |
|  | 3 | 1.09 | 0.94-1.26 | 1.13 | 0.98-1.30 |
|  | 4 | 1.06 | 0.91-1.23 | 1.21 | 1.05-1.39 |
|  | 5- least deprived | REF | - | REF | - |
