# Supplementary material for: Socioeconomic status and 30-day mortality after minor and major trauma: A retrospective analysis of the Trauma Audit and Research Network (TARN) dataset for England
Source: PLoS One. 2018 Dec 31;13(12):e0210226. doi: 10.1371/journal.pone.0210226 (PMC6312286; doi:10.1371/journal.pone.0210226)
Supplement: S4 Table — PMC- Comorbidity score; ISS- Injury Severity Score; IMD- Index of Multiple Deprivation. (DOCX) [file pone.0210226.s004.docx]

**S4 Table.**

|  |  | Minor | | Major | |
| --- | --- | --- | --- | --- | --- |
|  |  | OR | 95% CI | AOR | 95% CI |
| Age Group | 0-15 | REF | - | REF | - |
|  | 16-24 | 1.06 | 0.34-3.36 | 1.39 | 0.94-2.05 |
|  | 25-39 | 1.09 | 0.39-3.06 | 1.22 | 0.83-1.78 |
|  | 40-64 | 5.78 | 2.38-14.04 | 1.51 | 1.06-2.17 |
|  | 65-84 | 19.53 | 8.07-47.30 | 3.85 | 2.70-5.48 |
|  | 85+ | 47.79 | 19.72-115.76 | 8.28 | 5.78-11.84 |
| Sex | Female | REF | - | REF | - |
|  | Male | 1.23 | 1.13-1.34 | 1.04 | 0.95-1.14 |
| Injury Severity | ISS <9 | REF | - | REF | - |
|  | ISS 9-15 | 1.21 | 1.11-1.32 | 3.45 | 3.15-3.78 |
| Comorbidity score PMC | 0 | REF | - | REF | - |
|  | 1 to 5 | 2.68 | 2.39-2.99 | 1.96 | 1.76-2.18 |
|  | 6 to 10 | 4.28 | 3.76-4.87 | 2.90 | 2.53-3.33 |
|  | >10 | 8.02 | 6.68-9.64 | 4.23 | 3.41-5.25 |
| IMD Quintile | 1- most deprived | 1.26 | 1.11-1.43 | 1.01 | 0.88-1.15 |
|  | 2 | 1.12 | 0.98-1.27 | 1.03 | 0.90-1.18 |
|  | 3 | 1.12 | 0.99-1.27 | 1.07 | 0.93-1.22 |
|  | 4 | 1.02 | 0.89-1.16 | 1.15 | 1.00-1.32 |
|  | 5- least deprived | REF | - | REF | - |
